# X–ray absorption, phase and dark–field tomography through a beam tracking approach

**DOI:** 10.1038/srep16318

**Published:** 2015-11-06

**Authors:** Fabio A. Vittoria, Marco Endrizzi, Paul C. Diemoz, Anna Zamir, Ulrich H. Wagner, Christoph Rau, Ian K. Robinson, Alessandro Olivo

**Affiliations:** 1 Department of Medical Physics and Biomedical Engineering, University College London, Malet Place, Gower Street, WC1E 6BT London, United Kingdom; 2 Research Complex at Harwell, Harwell Oxford Campus, OX11 0FA Didcot, United Kingdom; 3 Diamond Light Source, Harwell Oxford Campus, OX11 0DE Didcot, United Kingdom; 4 London Centre for Nanotechnology, WC1H 0AH London, United Kingdom

## Abstract

We present a development of the beam–tracking approach that allows its implementation in computed tomography. One absorbing mask placed before the sample and a high resolution detector are used to track variations in the beam intensity distribution caused by the sample. Absorption, refraction, and dark–field are retrieved through a multi–Gaussian interpolation of the beam. Standard filtered back projection is used to reconstruct three dimensional maps of the real and imaginary part of the refractive index, and of the dark–field signal. While the method is here demonstrated using synchrotron radiation, its low coherence requirements suggest a possible implementation with laboratory sources.

X–ray phase contrast imaging (XPCi) is an established technique for the non–destructive analysis and visualization of specimens in a wide range of fields^1^, such as biomedical imaging, materials science, and others. In standard radiography, the contrast that enables the visualization of the specimens’ internal structures originates from the different x–ray absorption between features of interest and background. In XPCi, in addition to absorption, the phase shift experienced by x–ray wave fronts when travelling through matter is exploited, which leads to an increase in the final image contrast. This can be particularly important when low absorption materials are imaged, such as soft tissues in biomedical imaging. Different XPCi techniques have been developed over the years: Bonse-Hart interferometry^2^, propagation-based XPCi^3^, analyzer-based methods^4^, grating interferometry^5^, edge illumination^6^, and a series of alternative “single shot” methods^7^,^8^,^9^,^10^. Among these, some have demonstrated the capability to extract, alongside absorption and phase shift, the dark–field or ultra–small–angle x–ray scattering (USAXS) signal of the sample^10^,^11^,^12^,^13^. This latter signal is related to inhomogeneities in the sample refractive index on a scale smaller than the resolution of the imaging system, and can be used to discriminate materials with similar absorption/phase shift properties, but different microscopic internal structure (e.g. materials with a defined shape at a scale which is larger than the system resolution, but a different degree of homogeneity on a smaller, sub–resolution, scale). An additional advance in XPCi and USAXS is their implementation in tomography. Absorption, phase shift and scattering, in fact, can all be related to line integrals along the photon path of fundamental properties of the sample, and computed tomography (CT) can be used to reconstruct their three dimensional maps^11^,^14^.

We have recently shown how the edge illumination principle^6^,^15^ can be used in a beam tracking approach^16^,^17^ to reconstruct absorption, refraction and scattering. The main advantages of beam tracking over alternative XPCi approaches are that it does not require spatial or temporal coherence, can be adapted to work with laboratory sources, requires only one optical element placed before the sample, and can be used in a single–shot manner, reducing acquisition time and delivered dose, but at the cost of a reduced final resolution. Here we demonstrate its compatibility with implementation in a CT geometry, thus allowing the quantitative three–dimensional reconstruction of absorption, phase shift and scattering signals.

A scheme of the experimental setup is shown in Fig. 1. An incoming x–ray beam is shaped, through an absorbing mask, into a series of secondary, physically separated beamlets. While here a 1D–sensitive implementation (long, slit-shaped apertures) is used, extension to 2D mask structures is trivial, although potentially at the cost of a lower transmitted flux due to reduced mask open fractions. Each beamlet passes through the sample and, after a propagation distance z^p^, its intensity profile is recorded by a high resolution detector. The effect of the sample on a beamlet is to reduce its total intensity (due to absorption), change its direction (refraction), and increase its divergence (scattering). A multi–Gaussian interpolation^16^ of the intensity profile is used to extract the following parameters:


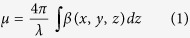



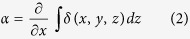



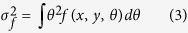


where *μ* is the attenuation coefficient, *α* the mean refraction angle, *β* the imaginary part of the sample complex refractive index, *δ* the difference from unity of the real part of the sample complex refractive index, *f* the scattering distribution, and *θ* the scattering angle. These three parameters are calculated from the variations, with respect to reference values obtained from an image without the sample, of the zero-th, first and second momentum of the beam intensity profile, which correspond to the total area, mean value, and variance of a Gaussian function, respectively. As described in^18^, 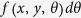 is the probability that a photon is scattered at an angle between *θ* and 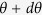 from the mean refraction angle *α* after passing through the sample. If we introduce the local scattering distribution 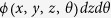 as the probability for a photon to be scattered at an angle between *θ* and *θ* + *dθ* from the local mean refraction angle after passing through the region of the sample between *z* and 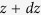, it is possible to write:


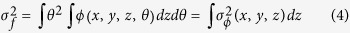


where 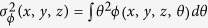. Equations 1, 2 and 4 express the retrieved signals as line integrals along the photon path of three physical properties of the sample. 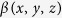 and 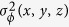 can be reconstructed using standard filtered back projection. 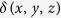, instead, can be reconstructed from *α* with a modified version of the filtered back projection, which adopts the Hilbert filter, instead of the ramp filter, to invert the derivative along *x* in the Fourier space.

## Results

**Figure 2:**
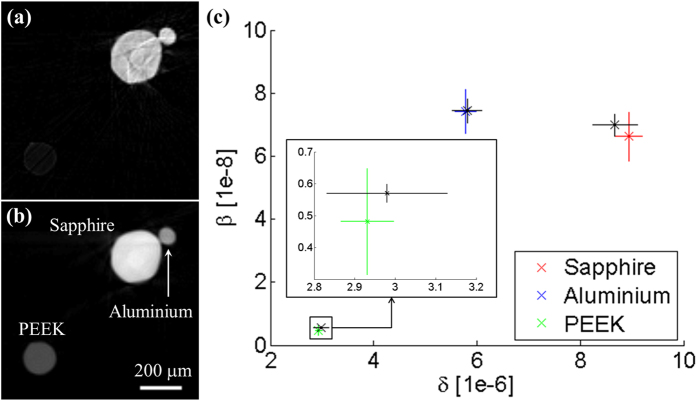
Reconstructed slices of (**a**) *β* and (**b**) *δ* from a test object made of three wires of different materials. In (**c**) the mean values calculated in the central region of each wire are compared with the theoretical ones (black). The error bars for the experimental data are equal to ±1 standard deviation, while an error of ±5% is assumed on the theoretical values to account for potential impurities and density variation. Resolution is reduced by approximately a factor of 2 compared to the intrinsic resolution of the system (≈10 *μ*m, equal to a mask aperture), due to the Gaussian filter applied to each projection to reduce noise in the final reconstruction (see text).

We first tested the quantitativeness of the method on a sample made of three cylindrical test objects of different, but known, materials: polyetheretherketone (PEEK), aluminium and sapphire. Results of the retrieval procedure and CT reconstruction are shown in Fig. 2. Figure 2(a,b) show a reconstructed slice of *β* and *δ*, respectively. Figure 2(c) shows a quantitative comparison between the retrieved values in the central region of each wire, and the theoretical ones. A good agreement is found for all the different materials, proving that the parameters extracted from the 3D images are quantitatively reliable.

**Figure 3:**
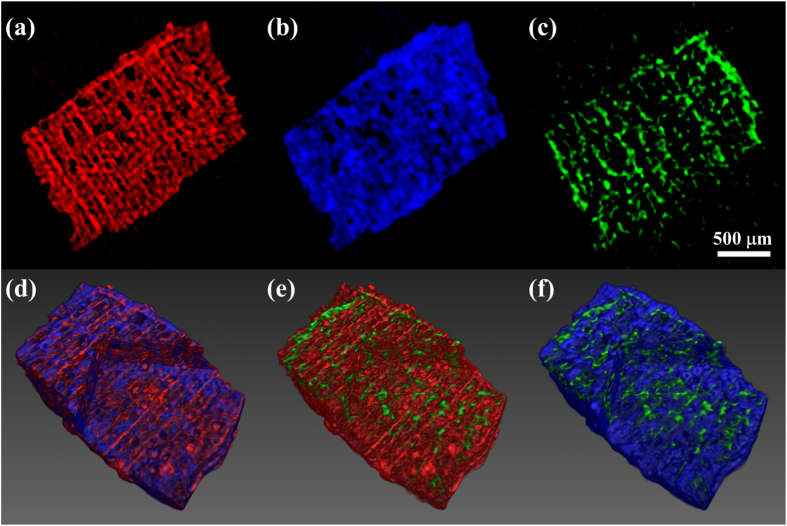
Reconstructed slices of *β* (**a**), *δ* (**b**), and 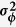 (**c**) from a wood sample. Volume rendering of *β* and *δ* (**d**), *β* and 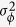 (**e**), *δ* and 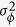 (**f**). The volume rendering has been sectioned to show three inner planes of the sample. Resolution is reduced by approximately a factor of 3, for (**a**,**b**), and 4, for (**c**), compared to the intrinsic resolution of the system (≈10 *μ*m, equal to a mask aperture), due to the Gaussian filter applied to each projection to reduce noise in the final reconstruction (see text).

The second sample we imaged was a piece of wood, which contains a complex internal structure arranged on different length scales. This sample was chosen because its sub–micrometric structures are expected to show a strong scattering signal, which might significantly distort the incoming beamlets. In this situation other methods^7^,^10^, based on the tracking of a speckle pattern, might present problems. The distortions induced by the sample on the reference pattern might, in fact, be so severe as to make it impossible to track the original speckle effectively. The advantage of our method, in this case, is to create a known, periodic reference pattern through a non-interferometric technique, whose variations can be tracked even for high values of the refraction and scattering signals. Figure 3(a–c) show reconstructed slices of *β*, *δ* and 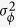, respectively, displayed with different colors. As expected, absorption and phase present similar features, in fact both these signals are ultimately related to the electron density of the sample. However, the contrast between different parts of the sample is locally different, and can be used to better identify regions of different composition within the sample. The scattering signal is not uniformly distributed within the sample. This signal, in fact, only comes from regions of the sample in which the refractive index is inhomogeneous on a scale smaller than the mask aperture. To better display the fact that these three channels provide complementary information about the sample, three volume renderings are shown in Fig. 3(d–f), where absorption, phase and scattering are superimposed in pairs.

## Discussion

We presented a method that enables performing quantitative x-ray phase–contrast and ultra–small–angle scattering computed tomography through a beam tracking approach. The method presents the advantages of a simple experimental setup, with only one optical element placed before the sample; absorption, refraction and scattering can be extracted from a single exposure of the sample, without the need to scan the optical element. A scan of the sample, instead, is needed to increase the final resolution and avoid possible aliasing artefacts, as will be explained in the next section. Additionally, the presented method does not rely on spatial and/or temporal coherence to generate contrast, suggesting the possibility of a future CT implementation with laboratory sources^17^. Indeed, the main requirement for this method, and for others based on edge illumination, is that the beamlets created by the absorbing mask remain physically separated to avoid ambiguity in the reconstruction; for a laboratory implementation this implies that the source size projected onto the detector plane needs to be smaller than the projected period of the mask. This effectively summarizes the spatial coherence requirements of the method^19^,^20^, while the even less restrictive ones on temporal coherence are discussed in^21^.

The quantitative accuracy of the method was experimentally tested on a sample consisting of three different materials of known composition and size, and a good agreement between the retrieved and the theoretical value of the sample refractive index was found. Finally, a CT reconstruction from a complex sample was presented, showing the robustness of the method against highly scattering materials, and that the three different signals can highlight different properties of the sample.

For this proof-of-concept experiment, each beam was tracked with a relatively large number of pixels, through a Gaussian fit. Future development will involve using masks with smaller aperture and period; this will result in a higher final resolution of the reconstructed images, and higher sensitivity to refraction and scattering signals. The assumption of a Gaussian profile was sufficiently accurate for the present experimental conditions, as the quantitative agreement in Fig. 2(c) demonstrates, however this might not always be true in the general case. The use of more refined fitting functions and of alternative retrieval method (e.g. through direct deconvolution of the beam profiles) will be investigated in future developments.

Finally, it should be noted that a simple free–space propagation setup, obtained by removing the absorbing mask, would allow a reduction in the total exposure time of about 1 order of magnitude. This, however, would result in a “mixed” image with absorption and phase effects superimposed, and in the loss of the dark-field signal. Optimizing the mask design to minimize exposure time, thus mitigating at least in part the required increase in exposure time, will be the subject of future research.

## Methods

The experiment was performed at the I13 (Coherence branch) beamline of the Diamond Synchrotron Radiation facility (Didcot, UK)^22^. A Si(111) crystal monochromator was used to select an x-ray energy of 9.7 keV. The mask is made of a gold layer electroplated on a graphite substrate, with aperture size and period of 10 *μ*m and 85 *μ*m, respectively. The detector consisted of a scintillator, a magnifying visible light optics and a CCD sensor, with effective pixel size of 1.1 *μ*m. Projections were acquired in the angular range [0° 180°], with 3 s exposure time per projection. For the test sample shown in Fig. 2, 181 projections were acquired with 1° step, and the detector was placed at a distance of 18.5 cm from the sample. For the wood sample in Fig. 3, 361 projections were acquired with 0.5° step, with sample-to-detector distance of 17.5 cm. For each angular position, a 10–step scan of the sample over one period of the x-ray mask was performed. In a single projection, in fact, parts of the sample covered by the absorbing septa of the mask are not illuminated and do not contribute to the signal. This results in a loss of resolution and in possible aliasing artefacts. The intrinsic resolution of our system, in fact, is determined by the mask aperture^23^. If, however, features in the sample vary slowly compared to the mask period, and a resolution equal to the mask period is acceptable, this scan can be avoided. Data were re–binned in the y direction in order to obtain a final voxel of similar size in the three directions (8.5 × 8.8 × 8.5 *μ*m^3^ in x, y and z, respectively). For the CT reconstruction, ramp and Hilbert filters were combined with a Gaussian filter, to reduce high–frequency noise in the reconstructed slices. The standard deviation of the Gaussian filter was chosen in relation to the noise level in the retrieved projections, and is equivalent to 8.5 *μ*m for the reconstructions in Fig. 2, 12.75 *μ*m for the reconstructions in Fig. 3(a,b), and 17 *μ*m for the reconstruction in Fig. 3(c).

## Additional Information

**How to cite this article**: Vittoria, F. A. *et al.* X–ray absorption, phase and dark–field tomography through a beam tracking approach. *Sci. Rep.* **5**, 16318; doi: 10.1038/srep16318 (2015).

## Figures and Tables

**Figure 1:**
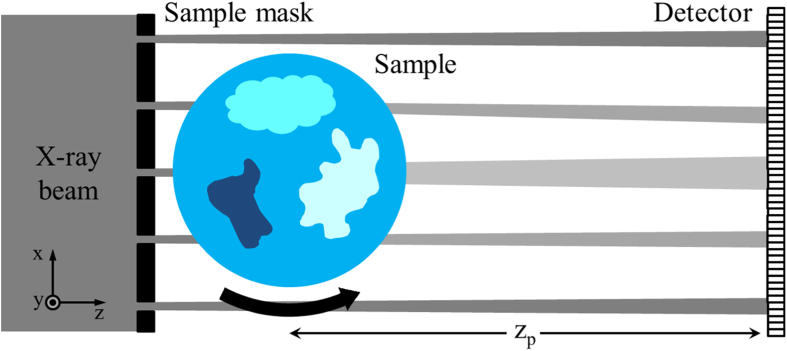
Schematic diagram of the experimental setup.
